# Utilisation of By-Product Phosphogypsum Through Extrusion-Based 3D Printing

**DOI:** 10.3390/ma17225570

**Published:** 2024-11-14

**Authors:** Maris Sinka, Danutė Vaičiukynienė, Dalia Nizevičienė, Alise Sapata, Ignacio Villalón Fornés, Vitoldas Vaitkevičius, Evaldas Šerelis

**Affiliations:** 1 Institute of Materials and Structures, Faculty of Civil Engineering, Riga Technical University, Kipsalas St. 6A, LV-1658 Riga, Latvia; maris.sinka@rtu.lv (M.S.); alise.sapata@rtu.lv (A.S.); 2 Building Materials and Structures Research Centre, Faculty of Civil Engineering and Architecture, Kaunas University of Technology, Studentu St. 48, LT-51367 Kaunas, Lithuania; ignacio.villalon@ktu.lt (I.V.F.); vitoldas.vaitkevicius@ktu.lt (V.V.); evaldas.serelis@ktu.lt (E.Š.); 3 Department of Electrical Power Systems, Faculty of Electrical and Electronics Engineering, Kaunas University of Technology, Studentu St. 48, LT-51367 Kaunas, Lithuania; dalia.nizeviciene@ktu.lt

**Keywords:** phosphogypsum, 3D printing, building materials, recycling materials

## Abstract

Phosphogypsum (PG) is a phosphate fertiliser by-product. This by-product has a low level of utilisation. Calcium sulphate is dominated in PG similar to gypsum and, therefore, has good binding properties (similar to natural gypsum). However, the presence of water-soluble phosphates and fluorides, an unwanted acidic impurity in PG, makes PG unsuitable for the manufacture of gypsum-based products. In this study, the binding material of PG (β-CaSO_4_·0.5H_2_O) was produced from β-CaSO_4_·2H_2_O by calcination. To neutralise the acidic PG impurities, 0.5 wt% quicklime was added to the PG. In the construction sector, 3D-printing technology is developing rapidly as this technology has many advantages. The current study is focused on creating a 3D-printable PG mixture. The 3D-printing paste was made using sand as the fine aggregate and a binder based on PG. The results obtained show that, despite the low degree of densification, 3D printing improves the mechanical properties of this material compared to cast samples. The 3D-printed specimens tested in [u] direction reached the highest compressive strength of 950 kPa. The cast specimens showed a 17% lower compressive strength of 810 kPa. The 3D-printed specimens tested in the [v] and [w] directions reached a compressive strength of 550 kPa and 710 kPa, respectively.

## 1. Introduction

### 1.1. The Problem of the Phosphogypsum

Phosphate rock is one of the most important mineral resources on Earth, since it is the prime matter from which phosphate fertilisers, such as ammonium phosphate fertilisers, are manufactured. However, during the production of phosphoric acid (an intermediate feedstock to produce the fertilisers, and mostly referred to as P_2_O_5_), an important by-product is manufactured: phosphogypsum. In 2023, the global production of P_2_O_5_ was 63.6 × 10^6^ tons and is expected to keep growing in the next years [1]. Considering the fact that for 1 ton of produced P_2_O_5_, about 5 tons of dry phosphogypsum (PG) are obtained [2], the estimated amount of produced PG is ~320 × 10^6^ tons.

Hence, it can be stated that PG is a relevant industrial solid waste at a global scale. The main problem carried by this by-product is its low rate of utilisation, since only 15 % is recycled [3], whereas the rest is stored in enormous stockpiles of discharged to water bodies so is damaging to the environment. Hence, there is an important interest of researchers in finding applications where PG could be utilised in large amounts. So far, the main fields are agriculture [4], soil stabilisation and road base construction (e.g., as a base layer in a parking lot [5] or as a sealing layer for roads [6]) and construction materials (as reviewed by various authors, such as Saadaoui et al. [7], who provide a general description of PG utilisation, or Dvorkin et al. [8], who focus on its application in mineral binders). Hence, building materials is a promising field, where PG can be used in two main ways: as an additive to Portland cement (OPC) and as a sole binding material itself. In the first case, PG could be included in OPC instead of ordinary gypsum, which is a well-known set retarder and strength accelerator [9]. Therefore, there is abundant investigation on the role of PG within OPC. For instance, Islam et al. [10] investigated the optimal PG content in OPC in terms of setting time, flow and compressive strength, whereas Akın and Sert [11] evaluated the performance of PG in respect to that of natural gypsum acting as OPC additive. However, as mentioned, PG can be used not only as an additive of other binders but as a binding material itself. The good binding properties of PG (similarly to natural gypsum), of its hemihydrate form, allows us to utilise PG to produce plaster, panels, tiles and even load-bearing products, such as bricks and blocks. So far, the main ways explored by academics to manufacture these PG products are rather the traditional ones: casting and press-forming. The latter has been proved by various researchers as an effective way to produce high-strength PG products, such as building blocks and bricks [12,13], tiles [14] and plasterboards [15]. However, innovative ways of creating PG products, such as three-dimensional (3D) printing, are still unexplored. Therefore, the current investigation focuses on the creation of PG products by 3D-printing technology.

### 1.2. 3D Printing

Three-dimensional printing, also known as “additive manufacturing” or “rapid prototyping”, is one of the cutting-edge technologies in the construction sector. As described by Gibson et al. [16], this technology consists of the manufacture of a physical solid object from a digital model, produced through three-dimensional Computer-Aided Design (3D CAD). The physical object is produced by adding the fresh material in relatively thin layers, which, joined together through curing or stacking, results in the final solid body. The main advantage of 3D printing is the fact that the solid can be produced without the necessity of process planning since it happens automatically [16].

Three-dimensional-printing technology is employed successfully in various industries, such as aerospace, automotive and healthcare industries [17]. In the construction sector, the first studies on 3D printing appeared in the last years of the last century by Pegna [18], and, from then, the interest on the topic has only increased, gaining a particular acceleration from the second half of the last decade [19]. El-Sayegh et al. [20] provide a deep discussion on the benefits, challenges and risks of the application of this technology in the construction industry. The main benefits are related to faster construction processes, lower costs (mainly due to the reduction in labour and materials) and more geometric freedom. Additionally, 3D printing is considered a sustainable process due to the possibility to easily employ low-impact materials (such as raw earth [21] and geopolymers [22]) and due to the reduction in the amount of waste in comparison to the conventional building methods [23].

On the other hand, 3D printing also attains some risks and challenges [20]. First, the complexity of some of the construction processes and materials, such as the reinforcement placing in reinforced concrete structures, is not easily integrated with concrete 3D printing. Moreover, scalability is a major challenge since the big sizes of the constructions made it difficult to implement what has been carried out in the laboratory at a lower scale. The lack of codes and regulations also hinders the implementation of these technologies. The mixtures of the printed materials (mainly concrete) should meet specific new requirements that are specific for the 3D-printing process, such as the pumpability, extrudability and buildability, and cannot be solely evaluated through traditional methods, such as slump test or setting time. In respect to the mechanical properties, it should be noted that the fact that the hardened printed material is anisotropic due to its layered nature makes it necessary to perform more tests than usual, such as the compressive strength test in three different directions and the strength test of the interlayer bond [19].

### 1.3. The Usage of Gypsum-Based Materials for 3D Printing

The main material employed for 3D printing is concrete, based on OPC binder [24,25]. However, the need to reduce the carbon footprint of the construction products has provoked the search for alternative cementitious binders, which are extensively reviewed by Peng and Unluer [25]. The main investigated alternative materials for 3D printing are geopolymers, MgO-based cements, aluminate cements, limestone-calcined clay-based cements and gypsum-based materials. Focusing on gypsum-based materials, it can be noticed that their particular properties such as low density, fire resistance and relatively high strength make them attractive materials for 3D printing [25]. However, the main drawback to consider is their low resistance to water, limiting their application for relatively dry conditions, such as internal walls, ceilings, claddings, sound absorbing panels and similar [26].

There are two main methods to print gypsum-based materials: powder bed binder jetting (PBBJ) and extrusion-based 3D printing [27]. The PBBJ technology consists of spreading a thin gypsum powder layer evenly in a surface (bed) and then injecting the adhesive only in the places where the gypsum material is supposed to harden. Then, a second powder layer is spread on the previous one, and the adhesive is again injected where needed. This process is repeated till the height of the final object is achieved. Once completed, the gypsum powder not bound by adhesive material is removed, and the final 3D-printed object (the glued part) remains. This type of 3D printing has been extensively investigated and is widely employed in industry. Aslan et al. [26] created porous cellular sound absorber gypsum panels using this technology, with a different configuration of the pore’s matrix, and determined the soundproof efficiency of each type of specimen. Kong et al. [28] investigated the usage of PBBJ technology to replicate natural rocks. The created gypsum specimens presented similar properties to fine-grained sandstone of low strength. On the other hand, one of the main problems of PBBJ technology is the tendency of gypsum powder to agglomerate, so hindering the smooth arrangement of the thin powder layers. Ma et al. [29] investigated the improvement of gypsum powder flowability by including 1.0 wt. % of hydrophobic nanosilica. It was found that the additive improved the quality of the gypsum powder bed, presenting a smoother surface and less undesirable voids.

Unlike the case of PBBJ printing method, research on extrusion-based 3D-gypsum-printing technology is not abundant. The reason for this is the quick setting time of gypsum, which, once mixed, begins to harden and becomes neither pumpable nor extrudable anymore. However, when the mixture is printed, a quick hardening is desired to avoid the collapse of the object when subsequent layers are printed on the top. Hence, the main problem for investigators is to find a suitable retarder/accelerator for the printing process of gypsum mixture. Huang et al. [27] investigated the influence of heat-induced accelerator on the setting time of gypsum plaster by applying different temperatures. It was discovered that the accelerator significantly reduced the setting time with temperatures higher than 40 °C. Additionally, the collapse ratio and shape retention index of the printed specimens also resulted satisfactorily. Gong et al. [30] investigated the extrusion process of recycled gypsum and combined several agents to improve the process: retarders (protein salt and polycarbonate superplasticiser), activation agent (sodium oxalate and dehydrate gypsum powder) and accelerator (sodium sulphate and potassium sulphate). The addition of retarders maintained the rheological properties by 40 min, whereas the subsequent addition of excitation components and accelerators enabled a quick hardening of 2–4 min after extrusion.

Hence, if research on the 3D printing of gypsum products by extrusion is rather seldom, the application of this technology with PG is inexistent. Therefore, the current research focuses on obtaining a 3D-printable PG mixture (with satisfactory buildability), which would present satisfactory mechanical properties in comparison to those exhibited by PG specimens produced by simple casting. The produced material is a sustainable solution in a double way since it combines the utilisation of the problematic by-product PG and the application of the sustainable 3D-printing process.

## 2. Materials and Methods

### 2.1. Initial Materials

Dihydrate PG (CaSO_4_·2H_2_O) is the by-product generated by JSC Lifosa (fertilisers plant) in Kėdainiai (Lithuania). Phosphogypsum formed as by-product from apatite from the Kovdor mine, Kola peninsula, Russia, during the production of phosphoric acid. First, PG was dried at 60 °C temperature until constant mass to remove the free water content. At this stage, the depicted loss on ignition (LOI) value was 18.92%, as typically exhibited by dihydrate calcium sulphate.

It must be reminded that dihydrate calcium sulphate is not a binding material. Therefore, it was processed through a thermal treatment (160 °C temperature for 1.5 h in amounts of 2 kg) to convert it into a hemihydrate (*HH*) phase (CaSO_4_·0.5H_2_O). In this case, after the treatment, the LOI value decreased to 6.7%, so confirming the HH nature of the material, which enables the binding properties.

The composition of HH-PG oxides determined by the X-ray fluorescence (XRF) method is presented in Table 1. It can be observed that the dominant compounds are calcium and sulphur oxides, the sum of which is 92.08%. Other oxides such as silicon, aluminium, magnesium, fluorine and phosphorus do not exceed 1%.

**Figure 1:**
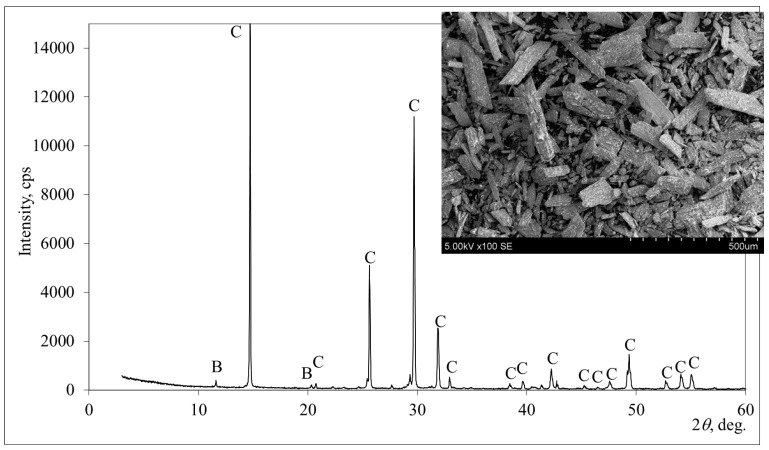
X-ray diffraction pattern and SEM image of phosphogypsum. Note: B is brushite CaPO_3_(OH)·2H_2_O (11-293), and C is basanite CaSO_4_·0.5H_2_O (81-1848).

The mineral composition of the HH-PG was determined from the X-ray diffraction (XRD) patterns given in Figure 1. As expected, basanite (CaSO_4_·0.5H_2_O) predominated in HH-PG. Traces of brushite (CaPO_3_(OH)·2H_2_O) were also depicted. No other compounds were identified. So, we can say that it is a low-contamination gypsum binder. This is also confirmed by scanning electron microscopy (SEM) analysis. The specific surface area was found to be 195.8 m^2^/kg of dry phosphogypsum powder. The specific surface area was found to be 195.8 m^2^/kg of dry phosphogypsum powder.

From the SEM image, it can be observed that HH-PG consists of flat prismatic crystals of different sizes. They are characteristic of hemihydrate calcium sulphate. Similar chemical composition of PG was detected by Cao et al. [31].

An amount of 0.5 wt. % quicklime additive was added to PG to neutralise the acidic PG impurities. The setting time and mechanical strength are close related to the neutralisation of acidic impurities. Several studies [32,33,34,35] have investigated the purification of PG from acidic impurities: acid leaching, alkali leaching, neutralisation (chemical purification) and separation, cyclone classification and thermal treatment (physical purification). CaO is one of the most effective and frequent solutions to neutralise these kinds of acidic impurities due to its high alkalinity.

Since the setting time for 3D-printing mixes is crucial for allowing suitable flowability and pumpability of the mixture, 0.1 wt. % setting retarder TARDA was added to PG to delay the setting time of the paste. The effect of CaO and retarder on the setting time of the binding material is provided in Table 2.

Moreover, a mid-range water reducing admixture—lignosulphonate-based plasticiser (Stachema, Riga, Latvia) was included at a dosage of 3 wt. % to reduce the water to PG binder (*W*/*PG*) ratio.

Additionally, the fine aggregate to produce the dry mixture consisted in sand, by Sakret Ltd. (Salaspils, Latvia), with particle sizes ranging from 0 to 2 mm.

All ingredients for used mixture PG-1 were proportioned by mass and are shown in Table 3.

#### Preparation of the Mixture

**Figure 2:**
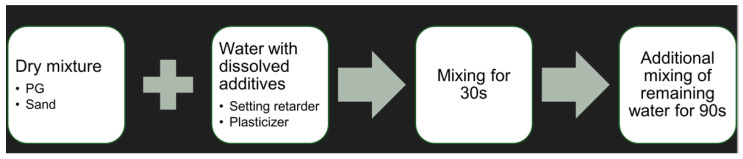
Flow diagram for 3D-printing mix preparation.

When mixing the mass for 3D printing, first, the setting retarder and plasticiser were dissolved in approximately half of the required water mass. Second, sand was added to the gypsum and homogenised through mixing. Then, the water/retarder/plasticiser mixture was added to the dry ingredients and mixed for 30 s. Subsequently, most of the remaining water was added and mixed for an additional 90 s (Figure 2). The mixing process was carried out using a portable mortar mixer, the RUBIMIX-9N, at a speed of 780 RPM.

Afterwards, the produced PG mixture was added to the printer. The printing process was performed using a gantry-type printer (custom-made) created at Riga Technical University in order to print various building materials, especially concrete. The printer frame allows for limited printing region dimensions of 1500 × 1000 × 1000 mm. The Repetier-Host software (Windows 2.3.2 version) by GmbH & Co. KG (Willich, Germany) was used to control the printer, and Simplify3D by Simplify3D Ltd. (Cincinnati, OH, USA) was used for slicing. The diameter of the printhead nozzle was 20 mm.

During the printing process, the mixture rapidly thickened and became almost unprintable. As a result, only one object was successfully printed. In the future, if 3D printing is carried out using this phosphogypsum, creating a mixture with an increased W/PG ratio could help solve this problem.

### 2.2. Experimental Techniques

#### 2.2.1. Chemical Composition and Microstructural Properties

The various chemical and microstructural properties of the initial materials and the printed specimens have been investigated through the following methods:

- Microstructure: SEM. Device: Hitachi S-3400N type II SEM microscope with an incorporated Bruker Quad 5040 detector (Tokyo, Japan).
- Elemental composition: XRF. Device: Bruker X-ray S8 Tiger WD spectrometer (Karlsruhe, Germany).
- Acidic water-soluble phosphate and fluoride content: colorimetric method. Devices: Hanna HI713 Checker HC^®^—Phosphate LR colorimeter (136); Hanna HI729 Checker HC^®^—Fluoride LR colorimeter (137) (Smithfield, VA, USA). Lithuanian technical conditions TS-21-154-86 was the reference according to which these analyses were performed [36].
- Mineral composition: XRD. Device: D8 Advance diffractometer, Bruker AXS (Karlsruhe Germany) (with geometry of Bragg–Brentano). Database for peak identification: PDF-2.
- Fineness of binding material: air permeability test (according to European standard EN 196-6) [37]. Device: Blaine air permeability apparatus.

#### 2.2.2. Rheological Properties and Setting Time

Buildability is the parameter that characterises this material’s ability to retain its shape after several layers have been deposited onto each other [38]. It is dependent on the deformation and flow of the used mortar, i.e., the rheology of material. To some extent rheology of mortar can be accessed via a consistence test of fresh mortar (by flow table), performed according to EN 1015-3 [39]. The W/PG ratio for normal consistency paste was also determined by EN 196-3 [40] standard. Additionally, initial and final setting time was determined using Vicat apparatus, according to EN 196-3.

#### 2.2.3. Testing Methods for the Mechanical Properties

##### Preparation of Hardened Specimens

Early strength was tested on cast samples after two different times: 60 min and 120 min after mixing. At the specified time, the sample was placed in the compression machine, and force was applied over its entire area. At least two specimens were tested at each time interval.

After a curing period of 7 days, the flexural and compressive strengths were determined for printed and cast specimens according to EN 1015-11 [41]. Density of both printed and cast samples was determined.

The fact that 3D printing is performed layer by layer determines the heterogenous nature of the produced specimens, which present different properties in different directions. Hence, according to Mechtcherine et al. [42], the orientations of the specimens and a specific coordinate system (*u*, *v*, *w*) were defined. Coordinate *u* is the printing path direction; *v* is perpendicular to *u* and remains in the printing plane; finally, *w* is perpendicular to the printing plane.

The orientation of the printed PG specimens for the compressive strength is given by one sole coordinate: the load direction for a normal compressive force. Meanwhile, the orientation of the specimens for flexural strength test is expressed through two coordinates: the first one represents the axis of rotation for the bending load; the second one corresponds to the longitudinal axis of the prismatic specimen.

**Figure 3:**
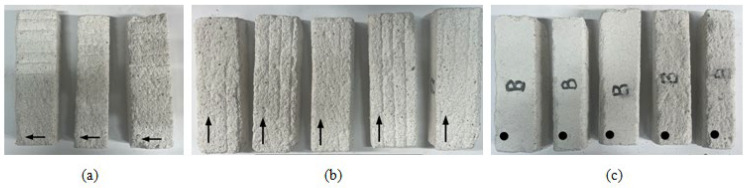
Test samples for three-point bending, top view: (**a**) orientation [*u.w*]; (**b**) orientation [*w.u*]; (**c**) orientation [*v.u*].

Prior testing, the print object (Figure 6) was cut using a circular saw to achieve regular prismatic-shaped test samples (Figure 3). After curing, the material exhibited brittleness and fragility. Additionally, the printed object had a relatively small layer width, making it impractical to cut samples with high precision. At least 3 specimens were prepared for each test direction.

Subsequently, each face of specimen was marked using the following symbols: an arrow for the *u,w* face (indicating the print direction), a cross mark for the *v,w* face and a dot for the *u,v* face (see Figure 3). 

##### Test Procedure

**Figure 4:**
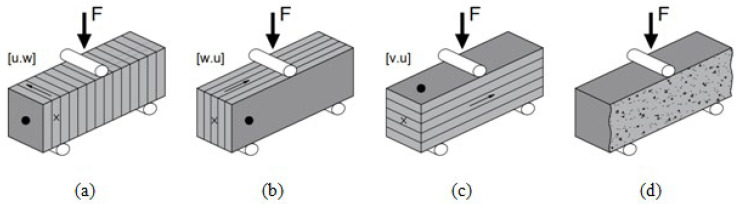
Three-point flexural strength test setup and sample orientation: (**a**) orientation [*u.w*]; (**b**) orientation [*w.u*]; (**c**) orientation [*v.u*]; (**d**) cast samples.

Three-point bending test was carried out for 3D-printed samples in three directions: [*u.w*], [*w.u*] and [*v.u*] as per setup (see Figure 4a–c) and for cast samples as well (see Figure 4d).

**Figure 5:**
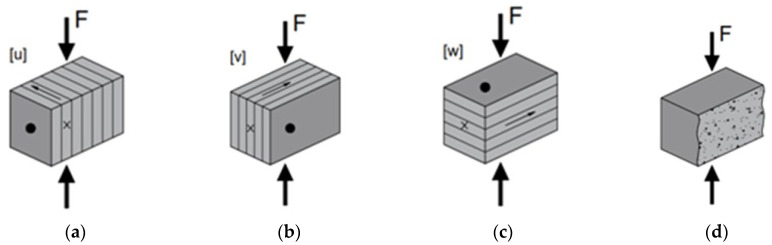
Compressive strength test setup and applied compression load perpendicular to top plane: (**a**) orientation [*u*]; (**b**) orientation [*v*]; (**c**) orientation [*w*]; (**d**) cast samples.

During the compressive strength tests, the samples were tested in three directions: [*u*], [*v*] and [*w*] (see Figure 5a–c) and for cast samples as well (see Figure 5d). Compressive strength tests were carried out using the broken halves of prisms used in flexural strength test.

## 3. Results and Discussion

As mentioned in the introductory section, 3D printing is a complex process, where both the properties of the fresh mixture and those of the hardened body are important. A suitable setting time and consistency of the mixture will allow a smooth printing process, whereas the density and strength of the hardened body will determine the aptness of the created building element. Hence, these properties are further discussed in the following sections.

### 3.1. Properties of the PG Mixture: Printability and Buildability

The suitability for 3D printing of the mixture can be evaluated through the results of the consistence test and setting time of the mixture, which are provided in Table 4. The results show that the diameter of the PG-1.1 mortar mixture spread was 205 mm after 5 min. As the time increased, the diameter gradually decreased, but, after 25 min, 192 mm of slump had occurred. The fresh state properties showed that this mixture is suitable for 3D printing. Similar results have been found by G. Bumanis et al. [43]. A mixture was developed for 3D printing with a slump of 138 mm after 38 min. The initial setting time was 36 min, and the final setting time was 66 min.

**Figure 6:**
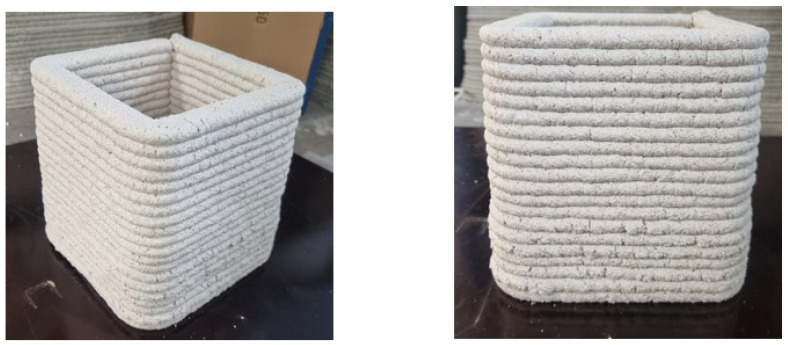
Square-shaped hollow print object immediately after printing.

Real 3D printing was performed with the PG mixture, and its buildability was studied. A square shaped hollow object (see Figure 6) consisting of 20 horizontal layers was printed at an extrusion rate of 67 mm/s.

The vertical layer interval time was 12 s, and the total print time was 1 min 50 s. Each layer was around 10 mm thick and around 30 mm wide. Neither horizontal nor vertical deformations of the printed structure were observed. Even though the added setting retarder slowed down the setting time, the bottom layer was the same thickness as the top layers. Some tearing of the surface finish was observed for the bottom layers. In general, the printability and buildability of the mass were satisfactory, although a slight increase in water could improve the surface quality.

### 3.2. Mechanical Properties

**Figure 7:**
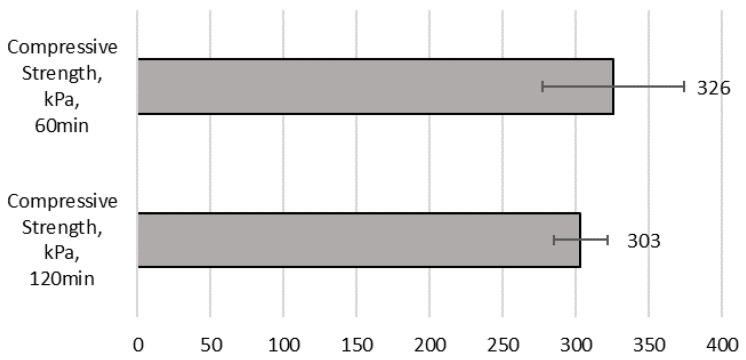
Test results—early compressive strength [kPa] of cast samples at 60 min and 120 min after mixing.

The compressive strength of cast samples provides valuable information about the hardening process of the mixture. Early compressive strength of not-dried cast samples tested at 60 min and 120 min after mixing reached 326 KPa and 303 KPa, respectively (see Figure 7), which is approximately 40% of the cast sample final strength after 7 days. It could be surprising that the 60 min strength value is higher than that of 120 min. However, this difference is between the error range so that is not relevant. The fresh state density of mixture was 1909 kg/m^3^.

**Figure 8:**
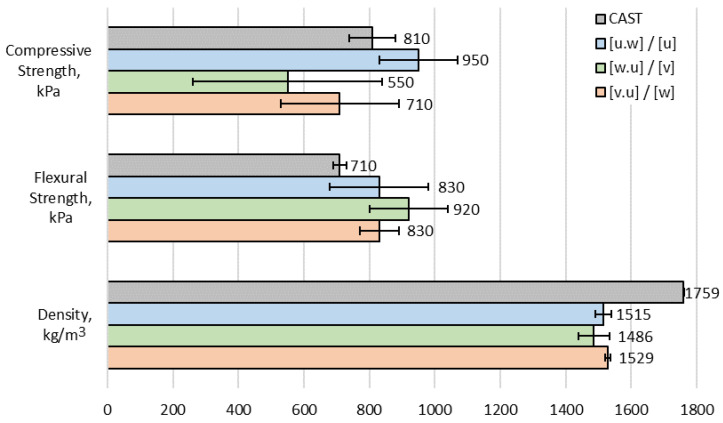
Test results—density, flexural and compressive strength of cast and 3D-printed specimens.

Figure 8 provides the density and flexural and compressive strength values of the specimens cured for 7 days. The results revealed that the printed specimens are remarkably less compact than the cast ones, presenting values of 1510 kg/m^3^ and 1759 kg/m^3^, respectively.

The results of 7-day flexural strength are also interesting (see Figure 8). Flexural strength is typically determined by the central bottom area of specimens, where the maximum tensile stress occurs [44]. Therefore, as compaction due to self-weight affects the bottom layer more than the top ones, 3D-printed samples in [*v*.*u*] direction usually exhibit higher flexural strength results than in other directions. However, during the testing, 3D-printed specimens tested in [*u*.*w*] and [*v*.*u*] direction showed identical flexural strength results of 830 kPa, whereas the specimens tested in [*w*.*u*] direction showed only 10% higher results, reaching 920 kPa. Hence, it can be observed that the print direction did not significantly affect flexural strength values and that the samples tested in [*v*.*u*] direction did not show higher flexural strength than in the other directions, as would be expected. This suggests that the degree of compaction of the bottom layers was not greater than that of the top ones. The previously mentioned observation that the bottom layers had the same thickness as the top ones also indicates that all layers were compacted equally. Moreover, the flexural strength of the cast specimens reached 710 kPa, which is 17% and 30% lower than for printed samples in [*u*.*w*], [*v*.*u*] and [*w*.*u*] directions, respectively. This result does not correlate well to the bulk density of printed and cast samples.

A possible explanation may be attributed to the lack of precision in the geometry of the printed specimens. However, this phenomenon also suggests that printed specimens with different numbers of layers exhibit an anisotropic character that depends on different directions. Lee et al. [45] stated that the extrusion process produces different porosities in the printed layers, which is important for the mechanical properties of the printed specimen. A similar explanation was used by Yang et al. [46]. In this study, the flexural and compressive strengths of the printed and cast specimens after 28 and 90 days of hydration are similar. Other researchers [47] have obtained a higher strength for the printed specimens than for the cast specimens. This may be due to compaction of the material during extrusion under pressure during the pumping process.

Finally, the compressive strengths were also evaluated. The 3D-printed specimens tested in [u] direction reached the highest compressive strength of 950 kPa (see Figure 8). The cast specimens showed a 17% lower compressive strength of 810 kPa. The 3D-printed specimens tested in the [v] and [w] directions reached a compressive strength of 550 kPa and 710 kPa, respectively. However, these results are not precise since their standard deviation values were relatively high. Most likely, the non-homogenous top and bottom planes of the compressed specimens were the reason behind the high standard deviation values.

Further research should investigate the challenges of scaling up 3D-printing technology from laboratory to industrial scale. This could help to understand how to implement the technology in practice. Materials being developed for 3D printing are relatively new, and their properties and behaviour during the printing process are not yet fully understood. Another challenge is the optimisation of the printing parameters to achieve the desired mechanical properties. Environmental factors such as air temperature, printing speed, layer height and infill are very important in the 3D-printing process. The quality control system is simply essential for many of the processes carried out by automated machines, as the cost of error can be very high. Three-dimensional printing is still a relatively expensive and time-consuming technology compared to traditional manufacturing methods. It is possible to reduce the cost of this technology by using secondary raw materials, printing in batches and automation. Finally, regulations and standards are underdeveloped in the industry and the market.

## 4. Conclusions

(1) In order to reduce the standard deviation of the compressive strength results in the future, specimens with a flat surface need to be prepared. Despite the challenging effects caused by the brittle material, the surface should be grinded after cutting, or even gypsum capping should be used to prepare the specimens.
(2) The obtained PG mortar showed low mechanical strength results. Therefore, it is suitable in construction only when combined with load-bearing structures or in cases when a structure that needs to hold only its own self-weight is produced. Such structures include permanent moulds and acoustic wall panels.
(3) The manufacturing of such elements is well suited for 3D-printing technology. It allows for the production of complex geometry without significant deviations, and this can be achieved an unlimited number of times. Additionally, the obtained results show that despite the low compaction degree, 3D printing increases the mechanical properties for this material in comparison to the cast specimens.
(4) The compaction degree of the printed element was low due to the low self-weight of the mixture. This might also affect not just mechanical strength but also durability, which was not investigated in this research. A higher degree of compaction could potentially be achieved by slightly increasing the W/PG ratio or by raising the extrusion rate.

## Figures and Tables

**Table 1:** Oxide composition of the HH-PG, %.

CaO	SO_3_	SiO_2_	Al_2_O_3_	MgO	F	Fe_2_O_3_	P_2_O_5_ *	P_2_O_5_ **	Other
38.45	53.33	0.37	0.13	0.04	0.14	0.03	0.82	0.40	6.70

*—total, **—water soluble.

**Table 2:** The influence of retarder on the setting time of PG.

Sample No.	Retarder wt. % *	Setting Times, min		*W/PG* Ratio
		Initial	Final	
PG-1	0.1	24	42	0.7
PG-2	0.2	40	75	0.7
PG-3	0.4	47	86	0.7
PG-4	1.0	Did not set after 2 h.	Did not set after 2 h.	0.7
PG-5	2.0	Did not set after 2 h.	Did not set after 2 h.	0.7

*—the amount of retarder was calculated from the amount of binder.

**Table 3:** Mix proportions of initial materials for 3D-printing paste (PG-1.1) and cast moulding (PG-1.1C), %.

Sample No.	Binding Material (PG + CaO)	Sand	Plasticiser *	Set Retarder *	*W/PG*
PG-1.1	40	60	1.2	0.4	0.68
PG-1.1C	40	60	1.2	0.4	0.68

*—the amount was calculated from binding material.

**Table 4:** Consistency and setting time of PG-1.1 mixture.

Property	Time, min	Spread Diameter, mm	Obtained Shape
After 15 jolts	5	205	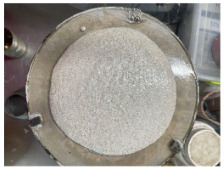
After 15 jolts	10	200	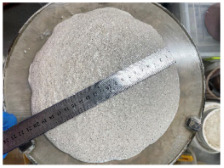
After 15 jolts	25	192	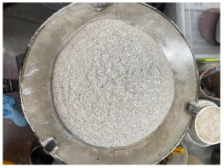

## Data Availability

The original contributions presented in the study are included in the article, further inquiries can be directed to the corresponding author/s.

## Abbreviations

OPC—ordinary Portland cement; PG—phosphogypsum; HH—hemihydrate; DH dihydrate; W—water; X-ray fluorescence (XRF); scanning electron microscopy (SEM); X-ray diffraction (XRD); loss on ignition (LOI); powder bed binder jetting (PBBJ); three-dimensional Computer-Aided Design (3D CAD); water to PG binder ratio (W/PG).
